# Impressive response to immunotherapy in a metastatic gastric cancer patient: could somatic copy number alterations help patient selection?

**DOI:** 10.1186/s40425-017-0291-9

**Published:** 2017-11-21

**Authors:** Gustavo dos Santos Fernandes, Daniel da Motta Girardi, Luiza Dib Batista Bugiato Faria, João Paulo Giacomini Bernardes, Renata de Almeida Coudry

**Affiliations:** 1 Department of Oncology, Hospital Sírio Libanês, Brasília, Brazil; 2 Department of Radiology, Hospital Sírio Libanês, Brasília, Brazil; 3 Department of Pathology, Hospital Sírio Libanês, São Paulo, Brazil

**Keywords:** Stomach neoplasms, Immunotherapy, Molecular targeted therapy, Somatic copy number alteration

## Abstract

**Background:**

Metastatic gastric cancer (GC) is an incurable and aggressive disease with a poor prognosis. Immunotherapy is an attractive approach for treating patients with cancer, and studies using immunotherapy have shown promising results in melanoma, kidney and non-small cell lung cancers, among others.

**Case presentation:**

We present a case of a 50-year-old woman with metastatic GC whose cancer had progressed after first-line chemotherapy and who received pembrolizumab as an experimental treatment. Molecular analyses showed that her tumor was negative for PD-L1 expression, contained microsatellite stability and several focal somatic copy number alterations. The patient experienced an almost complete response after eleven cycles of treatment. Her symptoms related to the disease disappeared, and the medication was well tolerated.

**Conclusions:**

Despite reports of promising responses in some patients, immunotherapy is not suitable for all patients; therefore, we explored the molecular characteristics that could explain the exceptional response and clinical benefits observed in our patient.

## Background

Gastric cancer (GC) is the fifth most common malignancy worldwide and the third leading cause of cancer-related death [1]. The Cancer Genome Atlas (TCGA) Project proposes a molecular classification of GC into four types, [2] although this classification has not yet been taken into account for clinical decision-making.

Metastatic disease has a dismal prognosis, with a median overall survival (OS) of approximately 11 months for HER-2-negative patients [3]. Recently, immunotherapy has emerged as one of the most promising strategies in cancer treatment, with outstanding results in many tumor types [4–6]. In GC, a phase 1b pembrolizumab trial have shown manageable toxicities and promising results [7]. Moreover, the use of nivolumab as a salvage treatment after second- or later-line chemotherapy has significantly improved OS, progression-free survival (PFS) and response rates compared to placebo [8].

In this paper, we report a patient with HER-2-negative metastatic GC who displayed a remarkable response to treatment with pembrolizumab as second-line therapy. We investigated the molecular characteristics that could be responsible for this successful outcome.

## Case presentation

A 50-year-old woman was diagnosed with locally advanced esophagogastric junction (EGJ) cancer in September 2014. Neoadjuvant chemotherapy with epirubicin, oxaliplatin and capecitabine was delivered for three cycles with significant diarrhea, peripheral neuropathy and asthenia. On October 13, 2014, she underwent a total gastrectomy. The pathology report evidenced a poor response (ypT3N2), so mFOLFOX6 adjuvant treatment was administered until January 2015. She remained on follow-up until February 2016, when metastatic disease in the lymph nodes was detected on computed tomography (CT) scans. At that time, mFOLFOX6 treatment has been initiated with palliative intent at another center, and she came to our institution for a second opinion. We decided to conduct a lymph node biopsy in order to confirm the diagnosis and perform molecular testing.

After the confirmed diagnosis of metastatic adenocarcinoma, we performed a commercially targeted next-generation sequencing (NGS) assay (FoundationOne from Foundation Medicine, Massachusetts, USA) in order to identify targetable molecular alterations. The assay revealed 18 somatic gene alterations and intermediate mutational load. Additionally, we analyzed the expression of PD-L1 and four mismatch repair (MMR) proteins (MLH1, MSH2, MSH6, and PMS2) by immunohistochemistry (IHC). We also measured Epstein Barr virus (EBV) in tumor samples by fluorescent in situ hybridization. The tumor samples were negative for PD-L1 expression, the MMR proteins were present, and EBV was not detected.

Due to the patient’s ability to tolerate mFOLFOX6, we decided to continue this treatment. After 9 cycles, progressive disease in the lymph nodes and liver was detected. We discussed that the standard treatment would be cytotoxic chemotherapy, preferably with paclitaxel and ramucirumab. She denied the use of this protocol due to the side effect of alopecia and the risk of worsening neuropathy.

We then offered to enroll the patient in the Keynote 181 clinical trial [9], which compares pembrolizumab to the investigator’s choice standard therapy. She was not willing to receive any standard chemotherapy and refused to participate in this clinical trial. However, she was very interested in immunotherapy. After tumor board discussion and careful conversation about the limited evidence of the efficacy of this treatment, we decided to offer her a treatment regimen with 200 mg pembrolizumab every three weeks.

**Fig. 1:**
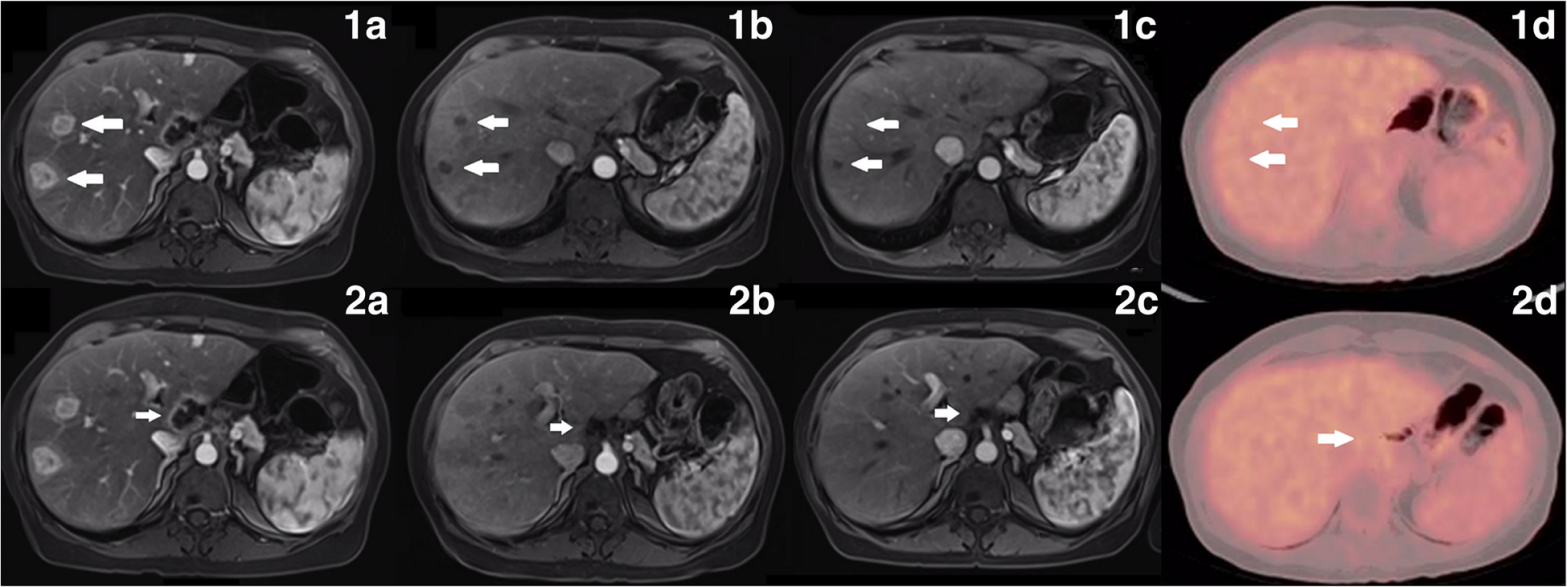
Columns **a**, **b** and **c** are magnetic resonance imaging and column **d** is a PET/CT imaging. White arrows in the first line show the reduction and complete remission of hepatic nodules. White arrows in the second line show the reduction and complete remission of a perihepatic lesion. **a**: Baseline images. **b**: Images after four treatment cycles. **c**: Images after eleven treatment cycles. **d**: Images after sixteen cycles

In September 2016, she started pembrolizumab. After 4 cycles of treatment, an abdomen magnetic resonance imaging scan revealed significant size reduction in all disease sites. New scans were performed after the 11th cycle of treatment. The retroperitoneal lymph nodes responded completely, and liver metastases and perigastric lesions were significantly reduced. A new positron emission tomography/computed tomography (PET/CT) was performed after 16 cycles of pembrolizumab and evidenced a complete remission of disease (Fig. 1). This patient has currently completed 12 months follow up and is receiving the 19th cycle of pembrolizumab without any side effects related to the treatment.

## Discussion and conclusion

Second-line treatment for GC usually consists of ramucirumab alone or in combination with paclitaxel or agents not used in first-line regimens, with limited results [10–12]. Immunotherapy has emerged as a promising strategy and has shown great benefits in many cancer types [4–6]. Although no single biomarker under investigation can accurately identify patients who are more likely to respond to immunotherapy, patients appear to be more responsive when their cancer harbors a high mutational burden [13, 14]. The expression of PD-1 and its ligands (PD-L1 and PD-L2) is frequently used to select patients for immunotherapy trials and appears to correlate with treatment response. However, not all trials select patients with increased PD-L1 expression, and responses are observed in tumors with low, high and negative PD-L1 expression [15, 16]. PD-L1 expression has been identified in approximately 40% of GC samples [17], and a recent phase 1b trial showed that 8 out of 39 patients with PD-L1-positive recurrent or metastatic GC or EGJ cancer responded to treatment [7]. Interesting results with nivolumab as salvage treatment for unselected patients based on PD-L1 results was also presented with median OS of 5.32 months with nivolumab versus 4.14 months with placebo (hazard ratio [HR], 0.63; *p* < 0.0001) [8].

Microsatellite instability (MSI) profiles also seem to predict clinical benefits of immunotherapy in gastrointestinal tumors. A phase 2 trial that evaluated patients with deficiencies in MMR (dMMR) proteins of the gastrointestinal system found that the immune-related objective response rate in patients with dMMR colorectal cancer was 40% versus 0% in patients with proficient MMR (pMMR) tumors [18].

In patients with non-small cell lung cancer with a high mutational load [more than 7 mutations found in the Foundation Medicine Cancer Gene Panel (FM-CGP)], a statistically significant anti-PD-1 treatment benefit was detected when compared to patients bearing tumors with a low mutational load (median PFS of 14.5 vs. 3.4 months, HR: 0.265, *P* = 0.005). These results suggest that the mutational load identified by cancer genome panels could represent a predictive clinical marker [19].

The tumor from our patient didn’t have expression of PD-L1 by IHC and was also proficient for MMR proteins. In addition, FM-CGP revealed that the tumors didn’t bear a high mutational load but instead contained somatic copy number alterations (SCNAs) in several genes. SCNAs are known to play a crucial role in carcinogenesis through the amplification of oncogenes or deletion of tumor suppressor genes [20]. A recent study investigated the different types of SCNAs (focal, arm and whole-chromosome) and their influence in two hallmarks of cancer: cell proliferation and immune evasion. The results showed that tumors with high levels of arm and whole-chromosome SCNAs tended to have reduced levels of cytotoxic immune infiltration, while focal SCNAs were more strongly associated with cell-cycle and proliferation signatures, implying distinct underlying mechanisms. The investigators also analyzed SCNA levels with regard to responses to anti-CTLA-4 inhibitors in metastatic melanoma patients. In this analysis, arm and whole-chromosome SCNAs were associated with worse outcomes and a poor prognosis [21]. In our case, FM-CGP showed a diploid tumor with focal SCNAs in several genes. This genomic landscape could be correlated with a less prominent immune evasion signature and, therefore, a positive response to immunotherapy. These are very preliminary data, and their application needs to be further investigated in the context of larger immunotherapy trials, including patients with other tumor types, such as GC, treated with anti-PD-L1/2 inhibitors.

Improved comprehension of the molecular alterations that drive carcinogenesis is important for precision medicine in terms of matching the right drugs to the right patients. Studies addressing molecular alterations in GC have the potential to guide pharmaceutical development and better select treatments for patients. An interesting study by Deng et al. sought to identify the most prevalent molecular alterations in GC using high-resolution single nucleotide polymorphism arrays to profile copy number alterations. These authors identified 22 recurrent focal SCNAs (13 amplified genes and 9 focally deleted genes) that include known targets such as ERBB2 but also novel genes [22]. The TCGA group proposed a molecular classification of GC into four subtypes. The first is categorized by EBV positivity, the second is characterized by MSI-high status, and the remaining two are distinguished by the presence or absence of extensive SCNAs [2]. These four GC groups have different molecular profiles that could potentially guide treatment. For example, PD-L1 expression is elevated in EBV-positive tumors, and a hypermutation status is more common in the MSI-high group [2]. Despite being highly relevant and widely recognized, this molecular classification currently has no practical application. Reports including molecular profiles in combination with clinical responses to novel therapies, such as immunotherapy, are important tools for understanding who can benefit from each type of treatment.

In conclusion, the selection of patients based solely on the PD-L1 expression and/or MI profile may exclude patients that might benefit from immunotherapy like our case. New and emerging knowledge about the role of SCNAs as predictors for patient responses to immunotherapy, along with the impressive response observed in our case, led us to hypothesize that the presence of focal SCNAs may be correlated with a less prominent immune evasion signature and, therefore, a better scenario for the use of immune checkpoint inhibitors. In order to clarify the molecular characteristics that could predict patient responses to immunotherapy, studies focusing on deciphering the molecular pathways of cancer are warranted, and molecular classification needs to be incorporated into future clinical trials.

## Abbreviations

CT: Computed tomography
dMMR: Deficiencies in mismatch repair
EBV: Measured Epstein Barr vírus
EGJ: Esophagogastric junction
FM-CGP: Foundation Medicine Cancer Gene Panel
GC: Gastric cancer
IHC: Immunohistochemistry
MMR: Mismatch repair
MSI: Microsatellite instability
NGS: Next-generation sequencing
OS: Overall survival
PET/CT: Positron emission tomography/computed tomography
PFS: Progression-free survival
pMMR: Proficient in mismatch repair
SCNAs: Somatic copy number alterations
TCGA: The Cancer Genome Atlas
